# Parental Evaluation of a Nurse Practitioner-Developed Pediatric Neurosurgery Website

**DOI:** 10.2196/resprot.5156

**Published:** 2016-04-12

**Authors:** Tina Kovacs Vogel, Manal Kleib, Sandra J Davidson, Shannon D Scott

**Affiliations:** ^1^ Faculty of Nursing University of Alberta Edmonton, AB Canada; ^2^ Alberta Health Services Edmonton, AB Canada

**Keywords:** Pediatric, neurosurgery, website, evaluation, parents, children, Google analytics, Internet health information needs, knowledge translation.

## Abstract

**Background:**

Parents often turn to the Internet to seek health information about their child’s diagnosis and condition. Information, support, and resources regarding pediatric neurosurgery are scarce, hard to find, and difficult to comprehend. To address this gap, a pediatric nurse practitioner designed a website called the Neurosurgery Kids Fund (NKF). Analyzing the legitimacy of the NKF website for parents seeking health information and fulfilling their social and resource needs is critical to the website’s future development and success.

**Objective:**

To explore parental usage of the NKF website, track visitor behavior, evaluate usability and design, establish ways to improve user experience, and identify ways to redesign the website. The aim of this study was to assess and evaluate whether a custom-designed health website could meet parents’ health information, support, and resource needs.

**Methods:**

A multimethod approach was used. Google Analytic usage reports were collected and analyzed for the period of April 23, 2013, to November 30, 2013. Fifty-two online questionnaires that targeted the website’s usability were collected between June 18, 2014, and July 30, 2014. Finally, a focus group was conducted on August 20, 2014, to explore parents’ perceptions and user experiences. Findings were analyzed using an inductive content analysis approach.

**Results:**

There were a total of 2998 sessions and 8818 page views, with 2.94 pages viewed per session, a 56.20% bounce rate, an average session duration of 2 minutes 24 seconds, and a 56.24% new sessions rate. Results from 52 eligible surveys included that the majority of NKF users were Caucasian (90%), females (92%), aged 36-45 years (48%), with a university or college degree or diploma (69%). Half plan to use the health information. Over half reported turning to the Internet for health information and spending 2 to 4 hours a day online. The most common reasons for using the NKF website were to (1) gather information about the 2 summer camps, (2) explore the Media Center tab, and (3) stay abreast of news and events supported by NKF. Parents were unanimous in reporting that the NKF website was pleasing in color and design, very easy to use and navigate, useful, and that they would continue to access it regularly.

**Conclusions:**

Parents perceive the NKF website to be useful and easy-to-use in meeting their health information needs, finding social support, and learning about resources relevant to their child. A custom-designed website can be used to augment parents’ health information needs by reinforcing, supplementing, and improving their understanding of their child’s medical needs.

##  Introduction

Children who require neurosurgery are a unique population with highly specialized medical needs. Information, support, and resources regarding pediatric neurosurgery are scarce, hard to find, and difficult to understand. Furthermore, connecting with other parents or caregivers whose children are also affected by neurosurgical conditions or illnesses can be even more difficult given the rarity of these diagnoses. Studying the impact and legitimacy of using a custom-designed website, the Neurosurgery Kids Fund (NKF) [1], to support parents’ health information needs—as well as their social and support needs—is imperative in bridging these gaps.

Searching for information on the Internet is a common first step for parents to gain knowledge about a child’s diagnosis, prognosis, treatment, and support options [2-4]. Once considered passive receivers of care, patients today are active consumers of health care who want to be involved in decision making, in managing their own health care, and in deciding how to mitigate risk factors and complications [5]. eHealth, the use of the Internet as a source of health information, offers many benefits, including data that complements the physician’s information, anonymous health information seeking, information exchange, community support, and empowerment in seeking help for and understanding of medical conditions [6]. People use online health information as a source of knowledge for many reasons. These reasons may include: getting immediate answers; learning about the diagnosis, treatment options, and prognosis; supplementing the physician’s information; finding support; and sharing with others who have similar experiences [2,6-11]. Parents of sick children report using the Internet to find health information because they worry about their child’s health, feel rushed and received limited guidance or advice from doctors, seek convenience and accessibility, and need to connect with others in similar situations [11].

Up until now, knowledge translation efforts have largely focused on ensuring that health care professionals use the latest research to inform their practice; however, initiatives that target health care consumers (eg, parents) can inform parental decision making, expectations, and shape their treatment outcomes [7,12,13]. Parents now have access to what was once privileged health information, potentially changing their understanding of their child’s medical condition, treatment options, medical decision-making, and relationships with health care providers [13]. Knowledge translation in child health is unique given family-centered care and the extent and level of parental involvement [14]. It is important to examine knowledge translation interventions such as websites that are developed specifically for parents to address health information needs and to provide resources and support tools (Appendix 1).

Crutzen, Roosjen, and Poelman argued that in contrast to self-reported exposure measures, tracking user behavior (eg, via dedicated software such as Google Analytics [15]) is independent of visitor’s memory, interpretation, or social desirability and when combined with qualitative methods, such as interviews, can yield a fuller and richer picture [16]. In Google Analytics, each visitor to the NKF website brings along his own set of data that can be collected, measured, analyzed, and reported and is an effective website evaluation tool guided by an analyst [17]. Similarly, Wilfert asserted that Google Analytics yields only statistical data but when paired with qualitative methods, a narrative unfolds with storylines including “how people got to the site, what they searched for when they were there, what they looked at, and what they did not” [18].

## Methods

### Design

This study used a multimethods approach including both quantitative and qualitative designs. Firstly, Google Analytics, a sophisticated Web analytics service, was employed to collect statistical data about NKF usage behavior. Secondly, a 20-question online survey questionnaire directed at parents about the usability of the NKF website was designed and collected using determinants of the technology acceptance model (TAM) [19,20]. Lastly, a focus group interview with parents about their experiences using the NKF website was conducted to augment the Google Analytics and online survey questionnaire data. The Google Analytics reports and online survey questionnaire results were used to inform and direct the focus group interview. Distinctions between usability and user experience are needed because the former is the ability of the user to use the website to carry out a task successfully (eg, used the NKF website to meet the health information needs addressed in the survey) and the latter takes a broader look at the individual’s interaction with the website, as well as the thoughts, perceptions, and experiences that results from that interaction (eg, reported during the focus group) [21]. Usage refers to the ways a website is used (eg, number of users, number of page views, time spent on page, etc). However, it is noteworthy that usability influences user experience.

Ethical approval was obtained from the University of Alberta Health Research Ethics Board. Consent was not required for Google Analytics as it is an embedded Web tool that collects anonymous grouped data. Participation in the online survey questionnaire and focus group interview was voluntary and signed consent was obtained for the focus group.

### Sampling

Recruitment was targeted at parents who have children, aged 0 to 16 years, who have undergone neurosurgery at the Stollery Children’s Hospital in Edmonton, Canada. Parents also had to be familiar with the NKF website for inclusion in the online survey questionnaire and focus group interview. Purposeful sampling was used in the focus group to ensure a breadth of age (for both the child and parent); parental education level; their usage of mobile devices, tablets, and/or computers; and their child’s neurosurgical diagnosis. Parents were excluded from the study if their English fluency prevented them from completing the survey or conversing in the focus group.

### Data Collection

Methodological triangulation was used and 4 sources of data collected: (1) Google Analytics reports, (2) online survey questionnaire results, (3) focus group interview with parents, and (4) field notes. Survey and focus group data were uploaded and kept secure in the Health Research Data Repository of the Faculty of Nursing at the University of Alberta. First, the Google Analytics data collection and analysis were conducted for the Web analytic and survey phases. With these results, revision of guiding questions for the focus group interview was performed. Google Analytics reports about NKF website usage were obtained from April 23, 2013, to November 30, 2013. This Web analytic tool uses client-sided data collection, called “page-tagging,” to collect raw data from the user’s browser. Google Analytics turns that raw data, or statistical numbers, into meaningful and usable information. Using a Web analytic tool, such as Google Analytics, removes bias and ensures speed, rigorous structure, and that an abundance of data can be collected [9,22].

The survey consisted of 20 multiple-choice and check-box questions with principals of the TAM underpinning the framing of the questions. The survey was developed after a review of the literature and piloted with 12 parents to ensure content validity and reliability. Developed by Davis, the TAM suggested perceived usefulness and perceived ease of use to be fundamental determinants of system use [19]. To summarize, a system, such as using a website, is more likely to be accepted and used if it is perceived to be useful and easy to use. Questions focused on the usability of the NKF website, namely: how parents seek health information; how and why they accessed the NKF website; whether their information, support, and resources needs were met online; if they discussed any health information found online with their health care provider; and the website’s perceived ease of use and usefulness. In addition, demographic data about the parents were collected including age, gender, ethnicity, location of residence, highest level of formal education, and computer usage.

A semistructured interview guide (Appendix 2) was used in the focus group, which lasted approximately 60 to 90 minutes and asked 4 parents about their experiences using the NKF website and its usability. The interview was recorded in real-time by a court reporter [23]. Benefits to using a court reporter to transcribe the focus group interview verbatim include increased data accuracy, timeliness, preserving confidentiality, and affordability [23]. Transcript-based data collection and analysis represents the most rigorous and time-intensive mode of analyzing focus group data [24]. The transcribed interview was cleaned by comparing the audio-recording with the transcript. Any identifiable information in the transcript was removed to preserve anonymity. Field notes were also obtained before, during, and after the interview to capture the context in which the data was collected. The field notes were reflected on during the data analysis to help situate when and how the responses were elicited (eg, nonverbal expressions or linguistic patterns).

### Data Analysis

Usage data for the NKF website was analyzed using the Audience, Acquisition, and Behavior reports from Google Analytics. The Audience report offers an overview of the time period selected, including the number of sessions logged, number of users, percentage of new sessions, number of page views, average session duration, bounce rates, number of pages per session, and location and languages used by the user. Reports about what type of browser and operating system were used and a mobile device overview and breakdown were analyzed. The Acquisition report examines how a user arrived at the website, which can reveal their purpose for visiting the website. Traffic analysis examines how well a website is supporting users who come to the site with specific information. Subheads of channels and mediums, all traffic sources, all referrals, and keywords were identified as metrics in this study. Lastly, the Behavior report offers information about what the user actually did when they arrived on the site; data about landing page, time spent on each page, the number of page views, and the percentage of exits and what page they exited from were analyzed.

Survey data were collected and entered into SPSS Statistics version 21 (IBM) from a text file and uploaded into a secure data repository. Data were verified for accuracy and cleaned. Seventy-four surveys were completed. Of those, 21 respondents were not parents or primary guardians of children with neurosurgery and 1 respondent indicated English was a second language with poor fluency; therefore, those surveys were excluded from this study. A total of 52 surveys were used in data analysis. Data were coded and descriptive data were computed for all variables.

The focus group interview data were analyzed using an inductive content analysis approach to address the purpose of the study [25]. The transcript was read as a whole several times and concepts, patterns, and themes were identified. With further immersion in the data, a coding system was developed and subsequent grouping and categorizing of the data into the recurring themes was performed. New codes and themes emerged throughout the analysis period and the data were continuously reexamined. The qualitative analysis software program NVivo 10 (QSR International) was used to assist with data management and analysis. In addition, a classic analysis strategy was used to make analysis a visual and concrete process [26].

Credibility was achieved in this study with methodological triangulation between the quantitative and qualitative data [27,28]. Both qualitative and quantitative methods were used together in an iterative process with neither method being weighted superior to another method [27]. Triangulation also allowed for the use of new research methods, Web analytics, to balance with the other methods in this study.

Credibility of the data was further achieved with transparency using an audit trail of all methodological processes. Reliability was achieved with the audit trail such that the results of this study could be replicated. Equivalence and internal consistency criterion were met because there was 1 researcher who was the only moderator and coder of the focus group data. Validity was enhanced with method triangulation because 2 or more methods demonstrated the same results and strict adherence to principles of qualitative research were followed. Field notes were reviewed to ensure that the findings were reflective of the focus group interview and not a reflection of any personal biases [10].

## Results

### Google Analytics Reports

#### Audience Report

For the first 6 months after the NKF website was launched, 2998 sessions and 1686 unique users were logged, with 56.27% (1687/2998) returning visitors. There were 8818 page views with an average of 2.94 pages viewed per session. The site bounce rate was 56.20% and the average session duration was 2 minutes 24 seconds. Using IP addresses to track and measure where a user is located, 90.23% users were from Canada (85.55% were from Alberta with 50.35% of them located in the Edmonton area). The remaining users were from: United States (172/2998, 5.74%), United Kingdom (35/2998, 1.17%), India (15/2998, 0.50%), Australia (8/2998, 0.27%), Ukraine (6/2998, 0.20%), and Philippines, Saudi Arabia, and South Africa (5/2998, 0.17%). The majority of users (2971/2998, 99.11%) viewed the NKF website in English. The remaining users accessed the website in French, German, Mandarin/Cantonese, or Arabic.

Users from the Philippines had the longest average session (4 minutes 5 seconds), followed by Canada (2 minutes 33 seconds) and the United Kingdom (1 minutes 28 seconds). Apple’s Safari browser was the most frequently used (1354/2998, 45.16%), followed by Internet Explorer (690/2998, 23.02%), Chrome (360/2998, 12.01%), Safari (app version; 241/2998, 8.04%), Mozilla Firefox (183/2998, 6.10%), and Android (117/2998, 3.90%). The majority of NKF website users (1628/2998, 54.30%) accessed the site using desktop or laptop computers. Mobile users accounted for 31.05% (931/2998) of all sessions and tablet users logged 14.64% (439/2998) of all sessions. Mobile users had the highest bounce rate with 68.74%, whereas computer and tablet users showed bounce rates of 50.06% and 52.39%, respectively.

#### Acquisition Report

Direct traffic accounted for 42.56% (1276/2998) of total visits to the NKF website. Organic search traffic using Google yielded 32.52% (975/2998) of users. Bing generated only 1.37% (41/2998) of users, and Yahoo brought only 1.03% (31/2998) of users to the NKF website.

Referral traffic accounted for 22.41% (672/2998) of the sessions. Average session duration for referral traffic was 2 minutes 5 seconds, with 2.82 pages viewed per session and a bounce rate of 54.76%. Of significance is that 24.67% (416/1686) of new users to the NKF site were acquired via referral sources. When mobile devices and tablets are combined, 70.09% (471/672) of all referrals were generated from Facebook, with a 50.41% bounce rate and an average of 3.25 pages viewed per session.

Since the NKF does not have any paid AdWords with any search engine company, only organic inbound keywords were analyzed. Organic search traffic yielded 1050 sessions, with an average session duration of 2 minutes 28 seconds, 3.03 pages viewed per session, and a 54.10% bounce rate. Variations of search terms “pediatric,” “neurosurgery,” “kids,” and/or “fund” accounted for 12 of the top 20 organic inbound keyword searches, or 24.86% (261/1050) of sessions. Of note, 48.48% (509/1050) of all sessions did not provide a keyword—this traffic arrived via a referral, used the URL directly, or had bookmarked the NKF website. The highest average session duration, using “www.neurosurgerykids.com” as a keyword, was 9 minutes 12 seconds, a significant outlier. The remaining top 8 keywords were related to specific fundraisers or events that were happening at that time. Only 1 medical term, “arachnoid cyst,” was included in the top 20 organic keyword searches. One search included the name of a pediatric neurosurgeon from the Stollery Children’s Hospital.

#### Behavior Report

The All Pages report for the NKF site illustrated a fairly typical distribution of the top 10 page views—the Homepage was the most viewed with 21.05%, followed by other pages that can be accessed from the Homepage with one-click buttons: About NKF (9.55%), Media Centre (7.62%), Join the Community (3.62%), Events (3.40%), Just for Kids (2.98%), Donate (2.88%), and Hope Stone (2.82%). The Media Centre’s subcategories of photographs and videos of children attending the NKF Camp or other events garnered the lowest bounce rate (23.53%) on the NKF website.

The NKF Homepage was the top-landing page with 52.97% (1588) of all sessions. Noteworthy are 3 landing pages that are buried further into the NKF site, which each garnered a number of sessions—arachnoid cyst (66), Just for Kids (47), and Hope Stone (45) . The NKF website did not have any significant outliers in the time spent on pages when combined with page views and unique page views. The range difference between page views and unique page views was 14%-38%. The Donate page attracted 216 page views, with 92 of those being unique, and users spent a lot of time there (2 minutes 37 seconds); however, the bounce rate was 85.87%.

### Online Survey Questionnaire

Demographic data and computer usage data were collected for 52 parents of children who have undergone neurosurgery. All participants resided in Alberta and a majority were Caucasian (47/52, 90%), female (48/52, 92%), aged 36-45 years (25/52, 48%), and had a university or college degree or diploma (36/52, 69%). Ninety-six percent of parents (50/52) reported accessing the Internet from home and 52% (27/52) spent approximately 2 to 4 hours a day online, with 21% (11/52) going online less than an hour a day and 25% (13/52) surfing the Internet for 5 or more hours a day.

A total of 42% (22/52) of parents reported that accessing health information on a computer as “very easy.” This was followed by 25% (13/52) and 21% (11/52) who said it was “somewhat easy” or “neither easy nor difficult,” respectively. Only 7.7% (4/52) of parents found accessing health information online to be “somewhat difficult”; however, no parents reported it being “very difficult.”

Several health information resources were reportedly used by parents. Ninety-eight percent of parents (51/52) reported relying on health care providers for their health information, followed by 77% (40/52) getting information from family and/or friends and 60% (31/52) going online to health websites. In addition, one-third (16/52, 31%) of the sample accessed medical journals and another third (18/52, 35%) reported favoring print media to supplement their health information search. One-fifth (19%, 10/52) of parents reported using TV or radio programming. Almost half (24/52, 46%) of the parents found reading health information on a computer compared to a book or pamphlet to be very easy, with 25% (13/52) saying it was somewhat easy, 21% (11/52) reporting it to be neither easy nor difficult, and 8% (4/52) stating it was somewhat difficult.

When parents were asked how they came to learn about the NKF website, 69% (36/52) responded that they learned about the NKF website from medical staff at a clinic or hospital visit, followed by 37% (19/52) hearing about it from family and/or friends, and 14% (7/52) came across it from an Internet search. Two respondents learned about the NKF site via Facebook or a local television or radio program.

Reasons why parents visited the NKF website included: to find more information about Camp Everest and L’il Everest Camp (67%, 35/52), to learn about upcoming media events and news related to the NKF (40%, 21/52), to check out the site in general (33%, 17/52), to find more health information about their child’s diagnosis or condition (23%, 12/52), to find social support and resources (21%, 11/52), to get their child a Hope Stone (17%, 9/52), and to make a donation (14%, 7/52).

Parents were also surveyed on how or if they planned to use the health information specifically found on the NKF website. Half of the parents (26/52) have discussed or plan to discuss the health information found on the NKF site with their child’s physician, nurse practitioner (NP), or other medical personnel involved in their child’s care. Six percent (3/52) of parents reported looking for health information from other sources. One-third (17/52) of the sample will discuss findings with family and friends and have contacted, or plan to contact, a support group. Further, survey results found that approximately 20% (10/52) stated that the health information found on the NKF website may influence future health decisions for their child and has improved their understanding of their child’s condition, surgery, or illness. The majority of parents (58%, 30/52) strongly or somewhat agreed that the health information found on the NKF website added to information from their child’s physician, NP, or other medical personnel, with 19 (37%) neither agreeing nor disagreeing and 2 (4%) strongly or somewhat disagreeing.

The survey examined parents’ perceptions about the ease of reading and understanding health information on the NKF website. Results found that approximately two-thirds of the parents (65%, 34/52) found the NKF website to be very easy to read and understand. Twenty-one percent (11/52) reported the website as “somewhat easy” followed by only 12% (6/52) who found it “neither easy nor difficult.” Only 1 parent (2%, 1/52) found the NKF website to be “somewhat difficult” to read and understand. Parents were also asked about their “favorite” part(s) of the NKF website and were allowed to give multiple responses (Table 1). Overwhelmingly, the NKF website was used to find more information about the 2 summer camps. Health information and Canadian content appealed to a large number of respondents. Supportive resources were reportedly also popular reasons to access the website. Please note percentages do not sum to 100% because of multiple responses.

### Focus Group

Of the 4 parents in the focus group, all were mothers with a child who had undergone neurosurgery a minimum of 2 years ago. The mothers’ ages ranged from 35 to 44 years, 2 had university or college degrees, and all had high school diplomas. Three self-reported their computer literacy as proficient and 1 described it as poor. All had familiarity with and used mobile phones and tablets regularly, and all of their children had attended Camp Everest. The focus group took place in a room with audiovisual equipment, and the NKF website was loaded and “surfed” throughout the session.

#### User Experience

Parents were asked to describe their experiences about where and how they began searching for information about their child’s neurosurgical diagnosis. All 4 of the parents strongly responded that they were reluctant to search online for mainly 2 reasons: (1) the timing of their child’s illness was a chaotic time and “when you’re in the hospital, it’s all very overwhelming” so searching online for information was not a priority. One parent reported not accessing it “until I was ready to go and do that,” further illustrating the impact of timing. This sentiment was further supported by the other parents in the focus group (Table 2).

**Table 1:** Parents’ favorite part(s) of the Neurosurgery Kids Fund website.

	Frequency n (%^a^)
Camp Everest and L’il Everest Camp information	41 (78.8)
News and events	34 (65.4)
Hope Stones	26 (50.0)
Social support and resources	23 (44.2)
Ease of use	22 (42.3)
Health information	21 (40.4)
Canadian content	19 (36.5)
Attractiveness, design, and layout	17 (32.7)
Donation information	15 (28.8)
Join the Community page	13 (25.0)

^a^Percentages do not sum to 100% because of multiple responses.

**Table 2:** Concepts and examples of parental experience using, or not using, the Internet.

Concept	Parent	Experience
Timing at acute phase of illness	Parent 2	“We were thrown into it … so you don’t have any time to do any research … so when that’s all happening and you’re bringing in a priest to give someone last rites, you’re not really thinking about a computer, see, and I would never read … when you had that thing up about trauma and stuff, I’ve already lived that nightmare, so I wouldn’t want to read that because I’ve already lived it, so I would never click that right now because I already know what it is (shaking her head, voice low and controlled, and pointing at the NKF screen).”
	Parent 3	“It was boom, boom, boom … everything happened at a very fast rate…. I remember [a nurse] saying going onto the NKF group, but I didn’t go home immediately and do it. I mean it, it sat there for a bit until I was ready to go and do that (arms gesturing dramatically in the air).”
Timing at chronic phase of illness	Parent 1	“Because he was born so early … we [searched online] later, before you had this [NKF website] set up.”
	Parent 2	“Because [our child] is pretty stable right at this moment.”
	Parent 3	“[Now] we’re okay; we’re in that stage of our lives where, you know, there’s nothing for us to do [like search online]. We have the support that we need.”
	Parent 4	“I think for us, just because [our child] has been stable for so, so long that really I go on here mostly about camp…. I know we’ve been blessed so far that—touch wood—you know, we’re not really going in for a lot of medical stuff.”
Influence of medical staff	Parent 2	“[Physician B] was very adamant. Don’t you dare touch that Internet, do not look at that—you listen to what I say, I’m the boss, and this is the way it’s going to run (other parents nodding).”
	Parent 3	“Well, I remember both [Physician A and Physician B] saying don’t Google it … we were directed by [a nurse]. And the doctors saying don’t go really anywhere (all other parents nodding).”
	Parent 4	“When we did research, it was basically only [Physician C].”

The second reason reported in the focus group for how or when these parents searched online for health information was that 3 of the 4 parents were advised by a physician or nurse to avoid using the Internet. Two mothers explain:

So when that’s all happening and you’re bringing in a priest to give someone last rites, you’re really not thinking about a computer. [Physician B] was very adamant, “Don’t you dare touch that Internet, do not look at it, do not— you listen to what I say, I’m the boss, and this is the way it’s going to run.”

Parent 2

I remember both [Physician A and Physician B] saying don’t Google it. [So later when searching online], I remember typing it in and feeling guilty about it. I just wanted the definition … I just wanted to know what the words meant….

Parent 3

Despite receiving cautionary warnings from their health care professionals, most of the parents reported going online eventually when their child was in stable health. Parents reported that they typed in a keyword, such as “VP shunt,” “cerebral palsy,” or “third ventriculostomy” into a browser. One parent described also using a “big encyclopedia book of brain and thinking, well, it doesn’t really have what I’m looking for.” One participant (with poor self-reported computer literacy skills) did not seek information on the Internet about her son’s diagnosis because “I’ve already lived that nightmare.”

All parents reported hearing about the NKF website by “word of mouth” from staff. All parents reported that the webpages were easy to navigate, “colorful, inviting, and joyful,” and even the non-tech parent said, “I’m not a computer person … I can just click that right there on the front, and that’s what I like.” All reported accessing the NKF website on their mobile phones without any difficulties, but when they wanted to read or explore the website at length, they used their home computers. One parent made many positive references to using her mobile phone to follow the NKF’s news and events via social media (eg, Twitter, Facebook). Difficulties on the website included the some technical errors (eg, not receiving a confirmation for registration into Camp Everest) and broken links (eg, brain tumor information page reported only an error message). Another parent agreed with the problem of broken links and also mentioned that some pages are not updated regularly. Parents described these 2 reasons for why the “medical conditions” pages were among the pages with the fewest page views. Findings from the Google Analytics data identified that the Community Resources page was infrequently viewed and used. Following up with parents on this identified some potential reasons for low page views and infrequent usage. Parents reported they did not know it existed, did not see the link, or had never visited the webpage. One parent questioned, “Is that the best name for it?” This led into discussion among the parents with a resolution that “Community Funding Support Resources” would more accurately describe the content.

Participants consistently used language of “safe” and “credible” when discussing the NKF website. The parents expressed feelings of “fear” and “mistrust” surrounding what they may find on the Internet and thus preferred to place their trust in their primary care providers (and the NKF website) to mediate the health information they received. This sense of legitimacy of the information on the NKF website is described by 2 parents:

This [NKF website] is a verifiable source … [said to be safe by other parents] … definitely … so they’ve kind of [sifted] out some of it so it isn’t this flukey, you know, therapy or surgery or doctor. Yeah, I felt safer … and if the doctors are telling parents not to Google it, if they are able to say, “Yeah, this is a verifiable source,” you know (other parents nodding in agreement).

Parent 1

[T]his is a safer place … definitely more … yeah, its’ credible … I had just the right information.… Here, I felt like, again, it’s been—someone’s already, you know, looked at it and thought, “This is right, this is perfect for what our parents are going to hear or read or see,” and I’d feel safer if it was through [the NKF website].

Parent 3

Parents also expressed fear surrounding accessing the Internet in search of health information and finding upsetting stories or poor outcomes:

I would never read … about trauma and stuff, I’ve already lived that nightmare, so I wouldn’t want to read that because I’ve already lived it, so I would never click that right now because I already know what it is.

Parent 2

I try and stay clear of reading other people’s stories or surgeries or mishaps or things like that or what went wrong, all that kind of things that you’re going to find.

Parent 3

#### Join the Community Tab

The NKF website is enabled with its own password-protected social network webpage called “Join the Community,” which is designed to function and serve as a forum or blog for parents, caregivers, and their children. By requiring them to register their minor children, parents give consent for their children to use it. Despite the Join the Community tab being on the home page, with one-click access, it was a seldom viewed and utilized feature of the NKF website. Parents were directed to the Join the Community tab for discussion and only 1 parent reported previously using it. The parent placed a message on the dashboard, never received a response, and thus abandoned it altogether. The parents cited reasons such as technical difficulties or unawareness as reasons for not using the Join the Community webpage.

Parents also described preferring to have an additional tab on the NKF homepage that is just for their children, “because I’d love for her to connect outside of camp with some of these kids.” When informed that this was the intended purpose of the Join the Community page, parents collectively identified hesitance in using it (Table 3).

**Table 3:** Parental opinion about having a blog or forum as a source of support parent-to-parent or just for the children.

Source of Support	Parent	Opinion
Blog or forum as a source of support for parent-to-parent	Parent 3	“If somebody was going through a similar situation, you could offer that I’ve been there, and you give … so even though it may not pertain to you, because right now [your daughter] is doing well and you already lived with it, as somebody else new comes, too, you could pop in and say … where you need to connect with others and chat.… (looking at parent 4)”
	Parent 4	“Yeah. I’d be very happy to be able to say to somebody, ‘Hey you can get through this.’ In fact, I went and did a talk at the [hospital] and it felt good to do it, sort of give some hope back, I guess … I know when I was going through it, I was pretty much a wreck.…”
Blog or forum as a source of support just for the children	Parent 1	“If you had a tab for adults and a tab for kids, I think would be better you know … just letting them go into their own site. I just think … if a parent is asking a question about something that maybe a parent doesn’t want their child to see, you know, like something went wrong … if the kids amongst themselves want to talk about, ‘hey, this is what I did,’ you know, that’s different than coming out of our fear as parents.… [If the kids have their own site] … so they’re not seeing the kind of … I think it would be better, you know?”
	Parent 3	“I think you have to get the kids involved with it, too. I showed him all of the pictures. I think the pictures really helped … but I was hoping that there could be a little bit more of that … because this is a safer place.”
	Parent 4	“I think I wonder about whether you want the kids—like, I kind of think sometimes the kids should almost have a different area than the adults for some of that stuff.”

The parents wanted a “safe” place to connect with other parents, or to ask a question, and they wanted their children, who are often too young or vulnerable to go online seeking peer support (eg, Facebook) to have a different tab to ensure a completely distinct and separate forum. One parent explains:

[My son] is absolutely terrified of needles, so if a parent is talking about “In this procedure you have to get this many needles” kind of thing … [my son is] not having to read that.… If the kids, amongst themselves, want to talk about, “hey, this is what I did,” that’s different than coming out of our fear as parents.

Parent 1

When prompted, the parents elaborated further:

If you had a tab for adults and a tab for kids … I think it would be better you know … just letting them go into their own site.… I just think—so the kids—if a parent is asking a question about something that maybe [another] parent doesn’t want their child to see, you know, like something went wrong … so they’re not seeing that kind of stuff.… I think would be better, you know?

Parent 1

I think you have to get the kids involved with it, too. I showed [my son] all of the pictures. I think the pictures really helped … but I was hoping that there could be a little bit more of that, because this is a safer place.

Parent 3

I think I wonder about whether you want the kids—like, I kind of think sometimes the kids should almost have a different area than the adults for some of that stuff.

Parent 4

##  Discussion

This study contributes to the literature demonstrating the legitimacy of using an online health website, Neurosurgery Kids Fund, for supporting parents seeking health information and fulfilling their social and resource needs. This study found that the health information found on the NKF website contributed and improved parents’ understanding of their child’s neurosurgical illness or condition. Themes not formally considered, such as how the timing during their child’s illness trajectory, parents’ fear of searching online, the context of what was being searched, and the influence of health care provider’s advice against online surfing were illuminated. The NKF website also serves as a single portal for meeting children’s and their parents’ support and resource needs in an accessible, attractive, and user friendly method that is easy to read and comprehend. Several studies have found that the Internet is a popular and efficient mode for distributing health information and offering social support because it is interactive, user controlled, offers anonymity, and is available around the clock [2,4-6,8,25]. In 2010, 8 out of 10 Canadian households (79%) had access to the Internet, with the second highest rate being in Alberta at 83% [29]. Among those, 70% of Canadians reported searching for medical or health-related information online [29].

### Parents’ Approaches to Searching the Internet

In this study we found that parents are increasingly accessing the Internet, particularly health websites, in search of health information, support, and resources. Hand et al [30] found that 83.4% of parents reported going online in search of information regarding their child’s health. DeLuca et al [2] and Kurup et al [31] found that parents are increasingly consulting other sources, mainly the Internet, even before visiting a health care professional. Parental usage of health websites for getting immediate answers; learning about the diagnosis, treatment options, and prognosis; adding to what the physician has explained; finding support groups; and sharing with others having similar experiences is well documented in the literature [2,5,11,13,25,30,32].

While the marrying of Web analytic, survey, and interview data created a picture of NKF website use, it can potentially lead to more confusion and questions. For example, both the Google Analytics and focus group data revealed that the Medical Conditions information pages (eg, hydrocephalus, achondroplasia) on the NKF website were less frequently visited compared to other pages (eg, Hope Stone, NKF News and Event , Media Centre). In contrast, the survey results reported that almost 25% of the parents visited the NKF website in search of health information and 40% of the surveyed parents rated it as one of their favorite parts. Inconsistencies in the findings can be perceived as a strength using methodological triangulation because it provides an opportunity to capture an unexpected new concept or theme [27]. Careful collection and insightful interpretation guided the concepts of timing and sample as potential reasons for the inconsistency in this study’s findings. The Google Analytics data was collected during the first 6 months after the website was launched and included data about anyone in the public accessing the site (eg, not parents with a sick child). For these visitors, digging deeper into the health information pages may not have held any relevance, and thus they avoided those pages. All the parents in the interview had children who had been diagnosed some time ago, described how they were more in the “chronic” phase in their child’s illness trajectory, and thus their health information needs were already met. In addition, the sample size was small and therefore these parents’ perspectives may not be representative of all NKF users. This example justifies why it is important to combine Google Analytics reports with other qualitative methods.

During the focus group, the parents explained not using the Internet to search for health information, primarily because their child’s neurosurgical diagnosis came during the acute phase of the illness—a time when life-saving decisions are needed in a very stressful situation. The parents in the focus group further described being overwhelmed and fearful, not wanting to relive the “nightmare,” and that the fear and uncertainty of their child’s health outweighed their desire to go online. Similarly, DeLuca et al found that parents wanted to learn about the medical condition, but were too anxious to directly search the Internet because of fear, further fueling their anxieties, or the potential for obsessing over negative content [2]. In contrast, Tuffrey and Finlay’s 2002 research involving parents of pediatric outpatients had a generally positive attitude toward the Internet and 88% felt that doctors should suggest suitable websites to parents [32]. Another study found that most people (72%) believe that all or most of the health information on the Internet is credible [33].

### Is Internet Health Information Seeking Context Dependent?

Gage and Panagakis’s 2012 study proposed that the type of health issue (eg, life-threatening condition versus routine health information) being confronted may be a critical dimension in understanding how, when, and why parents use the Internet as a source of health information [12]. A study involving patients before and after cardiac surgery, found that only 21% of the patients had used the Internet for health information [34]. Conversely, Chisolm found that health crises were consistent predictors of increased Internet use by patients for health information [35]. Knapp et al similarly stated that 76% of parents of children with life-threatening illness used the Internet for medical information [36]. Further, DeLuca et al found that nearly every parent acquired online information in the first hours and days after learning of the referral to a genetics specialist [2]. Despite the parents in our focus group describing cautious use of the Internet for neurosurgical information, 60% of the surveyed parents reported using websites for health information, which is comparable to the Canadian national average of 70% [29].

### Influence of Health Care Providers on Parents’ Health Information Seeking

Similar to the DeLuca et al 2012 study, some of the parents in this study were advised against seeking medical information on websites by their child’s health care providers [2]. The literature is replete with reasons why health care providers may be cautious about referring their patients to the Internet as a health resource: it may be inaccurate, unreliable, possibly even dangerous, has not been critically appraised (ie, peer reviewed), or may even be threatening to the image of the primary care provider [12,37,38]. However, as Nichols and Oermann stated, caution may well be advised when using the health information received on the Internet because of the unregulated nature of the medium, potentially giving way to obsolete and inaccurate information [39].

This study found that 98% of parents with a sick child prefer to receive specific health information from a trusted health care provider rather than on the Internet, and other studies found similar findings [2,6,7,40]. Similarly, Gage and Panagakis cited that during the highly emotional period following a diagnosis, parents may not want to be empowered through the Internet, but prefer to transfer some of the burden of decision-making to a trusted health care professional [12]. Knapp et al found that parents were more likely to trust information from a health care provider versus the information they located from Internet sources [36]. AlSaadi found that 68% of parents used health care providers as their main source of health information, although 79% of these same parents also reported using the Internet to gain information on their child’s health [8]. However, what is unique about the NKF website is that this information is created and provided by health care professionals.

### Parents’ Usage and Experiences Using the NKF Website

The results of this study showed that it is more common to seek health information on the NKF website among young to middle-aged Caucasian women who have higher levels of education and direct access to the Internet at home. Similar characteristics have been found in many other studies and have been dubbed the “digital divide” [6,7,31,41,42]. With the increasing ubiquity of mobile devices and tablets, this socioeconomic disparity may be negligible in the near future. Glynn et al dubbed the burgeoning use of mobile wireless communication devices as a subsection of eHealth called mHealth [6]. In this study, parents reported that being able to access the NKF website on their mobile devices or tablets (at their child’s bedside) day or night was a vital source of information and support.

In this study, 40% of the parents reported using the NKF website for health information. Despite 60% of the parents in this study reporting that the NKF health information added to their knowledge, only 20% reported that it may influence their medical decision making. Similarly, Glynn et al found that 29.1% of parents felt that the health information found online would influence the treatment decisions for their child [6]. In contrast, another study found that 68% of patients reported that the health information received online impacted their medical decision making [33]. However, 50% of the parents who received health information on the NKF website had discussed, or planned to discuss, their findings with their health care providers as compared to 34% of parents in another study [32]. Glynn et al similarly found that over half of the parents in their study had discussed, or intended to discuss, health information with their surgeon [6]. Only 6% of the parents in this study reported looking for health information elsewhere other than the NKF website. One of the parents in the focus group stated that the health information on the NKF website has been “verified” and is “just right for what our parents need.” Other studies found that some of the information available on the Internet is too technical in nature and not easily understood by the layman [2,8].

### Evaluating the NKF Website’s Usability

MacCulloch et al stated that website quality and presentation are critical elements in order for a website to be used effectively [43]. The findings demonstrated that the NKF website’s usability was evaluated to be: very easy to use, very easy to read and understand, informative, attractive, colorful and inviting, and easy to navigate. The parents reported the NKF website to have great “responsiveness,” meaning the dimensions were able to “flex” to the device (eg, mobile phone, tablet) being used—despite the Google Analytics reports indicating a high bounce rate for mobile phone users. When examined further, it was found that when parents used their mobile phones, it was mostly for quick fact finding, such as an address or contact information, or they were “on the go” and didn’t have time to graze on the NKF website.

Findings in this study suggest that the NKF website is congruent with the underpinning premise of TAM, which is that a website is more likely to be accepted and used by parents if they perceive it to be useful and easy to use [19,20]. However, some technical errors or broken links were identified by the parents; addressing these points and maintaining current updates can improve NKF usability and usage. Pew Internet and American Life found that 37% of users will leave a website if there are inadequate updates [33]. It is encouraging that 94% of the parents found what they were looking for on the NKF website, thus suggesting good usability. Ninety-two percent of parents plan to use the NKF website in the future, suggesting a good user experience.

### Parents Use of the Internet and NKF Website for Social Support and Resources

One of the most prevalent themes to emerge out of the collected data was the use of the NKF website for social support, connecting with peer parents, and resources. Plantin and Daneback found that using the Internet to establish connections with others in similar situations is of particular importance for parents whose children have serious medical conditions [7]. Google Analytics revealed that 6 of the top 7 landing pages were “social-related” pages. MacCulloch et al found a strong endorsement from the parents in their study for an online peer-based support network [43]. Similarly, Holtslander et al found in their study involving parents with diabetic children, that when parents can share experiences, it may more rapidly enable parents to achieve “normalization” following a life-altering diagnosis [44]. Results in this study revealed that 67% of parents reported using the NKF website for socially-related information and support—accessing Camp Everest and L’il Everest Camp information and parents “staying on top of things” by tracking the NKF’s News and Events (eg, fundraisers, parties, social gatherings)—and 70% arrived at the site via Facebook (another social gathering webpage).

### Study Limitations

There was only 1 focus group and the sample size was small (eg, 4 particiapants), however, parents brought a range and depth of experiences about having a child with neurosurgical concerns and their health information, support, and resource needs. The findings from the combined data of the Google Analytics, online survey questionnaire, focus group, and field notes were similar, indicating the main issues were identified (eg, theoretical saturation was met). The sample was predominantly mothers and, therefore, the relevance to fathers may be inappropriate. In future studies, health information should be clearly defined because it may mean different things to different people. Strict adherence to criteria for ensuring qualitative research trustworthiness increased confidence in the findings. There is a small potential for a margin of error in the Google Analytics data because all crawlers were granted access to the NKF website. In the future, to refine exploring only parents’ usage, a robot.txt file should be encrypted in the NKF website. The data were also collected over a relatively short period of time. Findings of website usage and experiences among parents of children undergoing neurosurgery may not be generalizable given the NKF website is targeted to the Edmonton, Alberta, region.

### Conclusions

There is a lack of research about the specific health information, support, and resource needs of parents with children undergoing neurosurgery. There is even less known about when they seek health information online, what health websites they are visiting, how useful the information was or was not, how e-literate they are, and, especially, why they are visiting the health websites that they do [14]. This study aimed to assess and evaluate whether a custom-designed health website could be used to meet parents’ health information, support, and resource needs. From this study, the majority of parents felt that the NKF website is credible, useful, and informative. Key findings that impacted whether parents sought online health information included the timing during the child’s illness, the context of the information being sought, and the impact of cautionary advice from their health care providers. However, after visiting the NKF website, many parents reported that the health information improved their understanding of their child’s condition, surgery, or illness. Other parents found the website to be a portal for joining the “NKF family” and for connecting with other parents for support and shared experiences.

Utilizing data and findings from this study, modifications to the NKF website will include expanding on specific health information and adding pictures related to neurosurgical diagnoses, equipment, treatment options, interventions, and prognoses provided by their own pediatric neurosurgeons, pediatric nurse practitioners, and allied health care providers involved in the care of this pediatric neurosurgical population. Blogs, video posts, and messaging designed by the health care team at the Stollery Children’s Hospital will be encouraged. These blogs, posts, and messages would reflect the 98% of parents who report relying on their direct health care providers for needed health information.

Additional modifications to the NKF website should be targeted at the support services and resources offered by the NKF including both L’il and Camp Everests, Hope Stones, and splitting the Join the Community pages between parents and children. These modifications would include more detailed explanation of the mission and purposes of the camps, eligibility, accommodations to the children’s specific health care needs, and qualifications of the camp counselors. More attention to the Just for Kids page will be outlined on the homepage explaining its purpose, target audience, and its safety measures to protect identity and confidentiality to the end users. Over 40% of parents also accessed the NKF website to stay abreast of news and events; therefore, keeping information updated, accurate, and informative will be stressed. From a technical standpoint, the NKF website should be monitored more closely and regularly for correct linkages and active pages.

The method of health care delivery is being transformed by the ubiquity of the Internet and the newly empowered, computer-literate public is making a claim in becoming partners in managing their own health. Such changes have the potential to bring about positive outcomes, such as improved medical decision making, increased efficiency in the clinic or hospital appointment, and strengthening the relationship between primary health care providers and the patient’s parents. The time is now for the health care profession to respond to the “Internet-informed” parent by guiding them to reliable health information websites, giving them a “health website prescription,” and collaborating with them in obtaining and analyzing the information received.

## Appendix

Multimedia Appendix 1 The NFK website was developed specifically for parents to address health information needs and to provide resources and support tools.

Multimedia Appendix 2 Guiding Focus Group Interview Questions.

## Abbreviations

JMIR: Journal of Medical Internet Research
NKF: Neurosurgery Kids Fund
TAM: technology acceptance model
