# Supplementary material for: Parental Evaluation of a Nurse Practitioner-Developed Pediatric Neurosurgery Website
Source: JMIR Res Protoc. 2016 Apr 12;5(2):e55. doi: 10.2196/resprot.5156 (PMC4846784; doi:10.2196/resprot.5156)
Supplement: Multimedia Appendix 1 [file resprot_v5i2e55_app1.pdf]

## Appendix 1 – Supplementary Figures

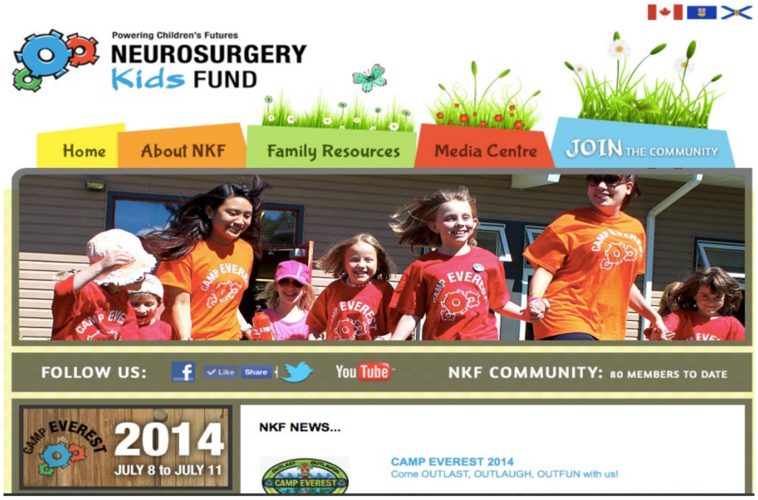


Figure S1. Homepage of the Neurosurgery Kids Fund website.

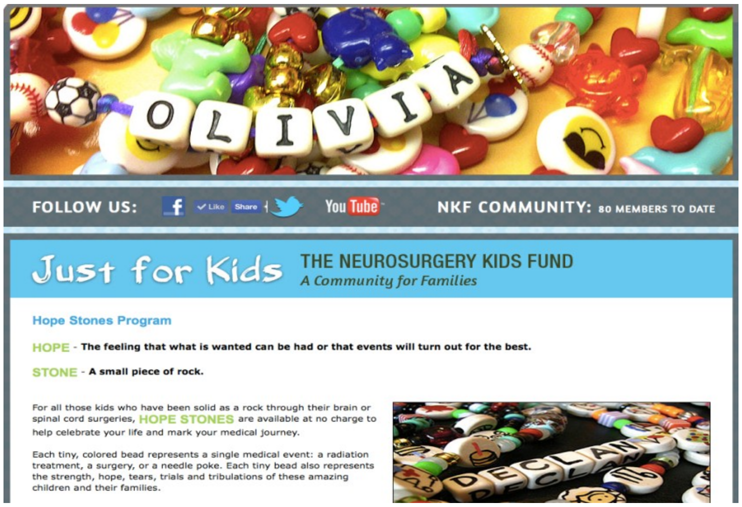


Figure S2: Screenshot about the Hope Stones, bead program.

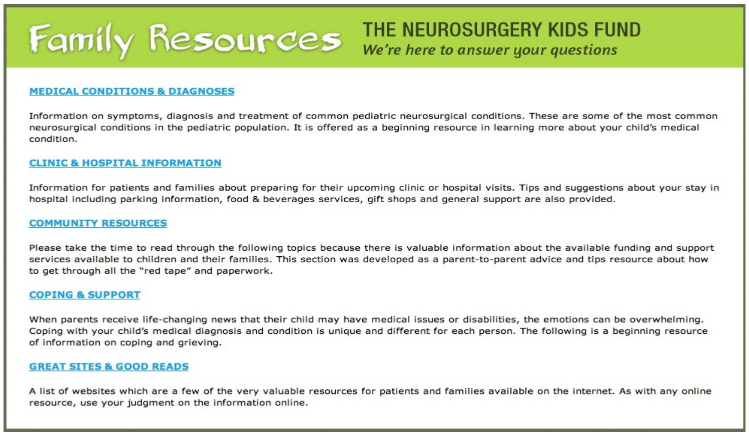


Figure S3: Screenshot of Family Resource page.

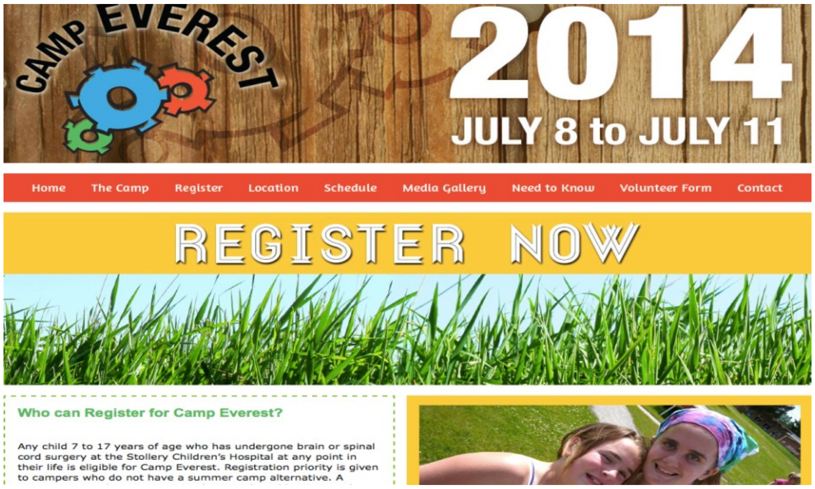


Figure S4: Screenshot of Camp Everest page.

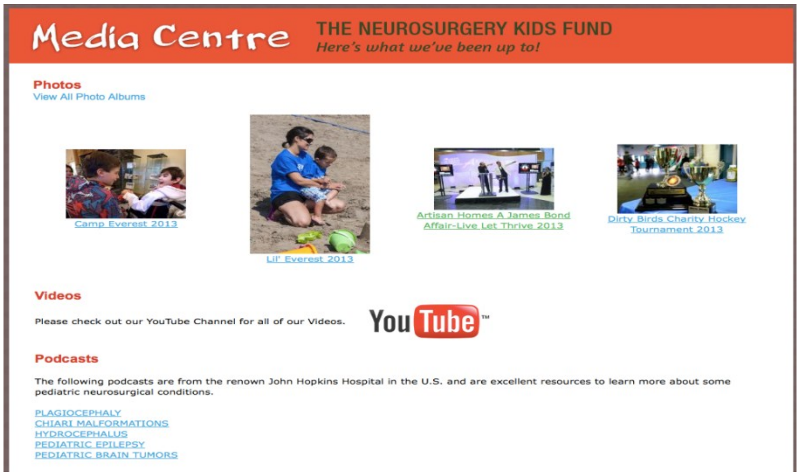


Figure S5: Screenshot of the Media Centre.

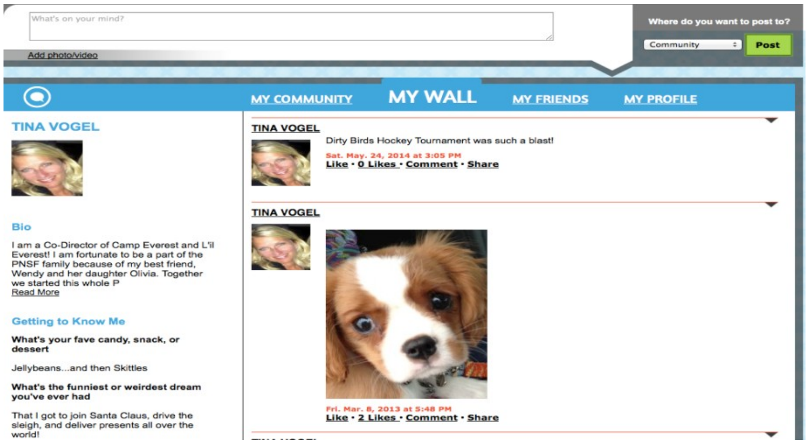


Figure S6: Screenshot of the Join the Community.
