# Supplementary material for: Parental Evaluation of a Nurse Practitioner-Developed Pediatric Neurosurgery Website
Source: JMIR Res Protoc. 2016 Apr 12;5(2):e55. doi: 10.2196/resprot.5156 (PMC4846784; doi:10.2196/resprot.5156)
Supplement: Multimedia Appendix 2 [file resprot_v5i2e55_app2.pdf]

Appendix 2 - Guiding Focus Group Interview Questions

I have collected statistics about how people are using the NKF website. Those numbers have helped me get an idea of ***how*** people are using the website but it doesn't tell me ***why*** or ***what*** people are using the website for. This is what I'd like to ask you about today. To begin:

1. Tell me about your experience as parents of children who have had neurosurgery looking for information? What kind of information are you looking for?

2. What prompted you to use the Neurosurgery Kids Fund website?

3. Tell me about your experience with using the Neurosurgery Kids Fund website?
    a. What did you think of the website in general? Design?
    b. How did you find getting around the different pages?
    c. What were the things you liked or found useful about the website? Tell me about them.
    d. Was anything on the website not useful or something about it you didn't like?
    e. If you could suggest for improvements on the website, what would that be?

4. Has using the NKF website influenced you as a parent of a child with health concerns?

5. I am going to share with you some of the statistics about our website. I would like you to share your perception about them.
    a. Why do you think the Medical Conditions pages were seldom viewed?
    b. Why do you think the numbers show that a lot of traffic is going to Camp Everest? Poor traffic to Join the Community?
